# Innate Immune Response in SARS-CoV-2 Infection

**DOI:** 10.3390/microorganisms10030501

**Published:** 2022-02-23

**Authors:** Giovanna Schiuma, Silvia Beltrami, Daria Bortolotti, Sabrina Rizzo, Roberta Rizzo

**Affiliations:** Department of Chemical, Pharmaceutical and Agricultural Sciences, University of Ferrara, 44121 Ferrara, Italy; giovanna.schiuma@unife.it (G.S.); silvia.beltrami@unife.it (S.B.); sabrina.rizzo@unife.it (S.R.); rbr@unife.it (R.R.)

**Keywords:** SARS-CoV-2, immunity, innate immune cells, immune-evasion, natural killer cells

## Abstract

An efficient host immune response is crucial in controlling viral infections. Despite most studies focused on the implication of T and B cell response in COVID-19 (Corona Virus Disease-19) patients or in their activation after vaccination against SARS-CoV-2, host innate immune response has raised even more interest as well. In fact, innate immunity, including Natural Killer (NK) cells, monocytes/macrophages and neutrophils, represent the first line of defense against the virus and it is essential to determine the correct activation of an efficient and specific acquired immune response. In this perspective, we will report an overview on the main findings concerning SARS-CoV-2 interaction with innate host immune system, in correlation with pathogenesis and viral immune escape mechanisms.

## 1. Introduction

SARS-CoV-2 is a positive-sense single-stranded RNA (ssRNA) virus that has emerged around the end of December 2019, in Wuhan, Hubei-China, associated with a severe respiratory syndrome named COVID-19 (Corona Virus Disease-19) and declared pandemic in March 2020. 

SARS-CoV-2 can infect target cells through the interaction between its structural glycoprotein Spike (S) protein [1] and ACE-2 (Angiotensin Converting Enzyme-2) receptor. The wide tissue expression of ACE-2 determines SARS-CoV-2 tropism for several organs and explains the variety of symptoms associated to the infection [1]. In addition to ACE-2, other molecules, such as CD147 and neuropilin 1 (NRP1), have been identified as possible co-receptors able to enhance the ability of SARS-CoV-2 to enter human cells [2,3]. Both CD147 and NRP1 are widely distributed in body tissues and play diverse physiological as well as pathological and therapeutic roles in different clinical conditions, including COVID-19 [4,5], involving host immune system activation [6,7].

Different findings have reported SARS-CoV-2 ability, and particularly of its Spike protein, to interfere with host immune activation [8].

During SARS-CoV-2 infection, both innate and adaptive immune system are engaged [9]. The innate immune system, referred to Natural Killer (NK) cells, monocytes/macrophages and dendritic (DC) cells, is the first to be recruited, followed by T and B lymphocytes responses, responsible for the specific pathogen recognition and for the establishment of the immunological memory.

SARS-CoV-2 infection has been described as associated to peculiar effects involving both immune components, strongly related to COVID-19 symptoms such as the “cytokine storm” condition [10]. Even if most studies focused on T and B cells response to SARS-CoV-2 infection, and the engagement of immunological memory raised after natural infection or vaccination, innate immunity plays a crucial role in COVID-19 onset, as the first line of defense against SARS-CoV-2 infection [11].

**Figure 1:**
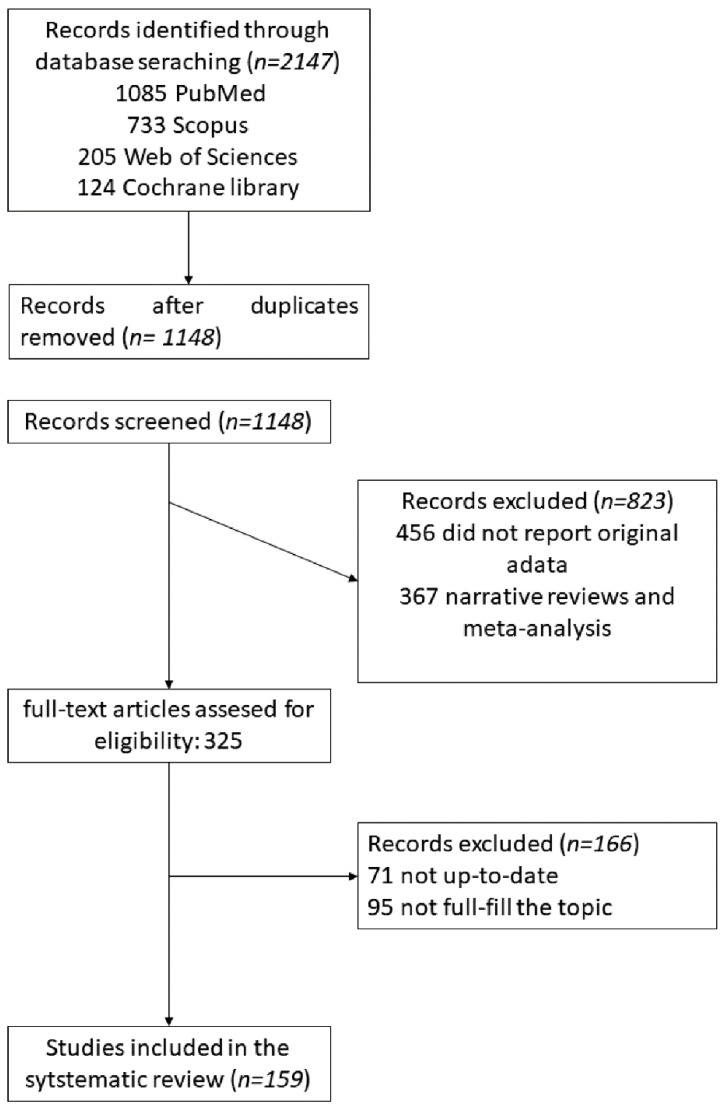
Data selection following eligibility criteria accordingly to the reviewed topic.

In this perspective, we will review the main current data referred to SARS-CoV-2 interaction with innate host immune system, in correlation with pathogenesis and viral immune escape mechanisms. Data were selected follow eligibility criteria accordingly to the reviewed topic, as reported in Section 2 (Figure 1).

## 2. Materials and Methods

### 2.1. Search Strategy

We used a set of electronic databases (Medline/PubMed, Scopus, Web of Sciences (WOS), Cochrane library) for a systematic searched until January 2022 using MeSH keywords/terms: “COVID-19,” “2019 novel coronavirus,” “RNA sensors” “inflammasome”, “interferon”, immune cells”, “innate immunity”, “Natural killer cells”, “NETs”, “monocytes”, “macrophages”, “neutrophils”, “cytokine storm”, “immune cross-talk”, “immune-escape”, “antiviral effect”. We applied no date or language restriction. We followed the Preferred Reporting Items for the Systematic Review and Meta-Analysis (PRISMA) statement [12]. 

### 2.2. Study Selection

We selected two independent reviewers to performed title-abstract screening on all selected studies, then the full-text of the selected articles were reviewed. In cases of duplicate information from the same patient, the data were checked and combined, but only considered as a single case.

### 2.3. Inclusion Criteria

Studies reporting innate immune response, as well as COVID-19 were selected. Publications were selected using specific keywords (“COVID-19,” “2019 novel coronavirus,” “RNA sensors” “inflammasome”, “interferon”, immune cells”, “innate immunity”, “Natural killer cells”, “NETs”, “monocytes”, “macrophages”, “neutrophils”, “cytokine storm”, “immune cross-talk”, “immune-escape”, “anti-viral effect”) and MeSH Advanced Search Builder. Articles were filtered also according to the date of publication (not older than late 2020–2021) and to fulfill the topic of this perspective.

### 2.4. Exclusion Criteria

Studies which were just case reports, and commentaries were excluded. Moreover, publication without DOI (e.g., conference abstracts and clinical trials) were excluded.

### 2.5. Data Extraction

The extraction of the data from included studies was performed by two reviewers separately, considering key characteristics including publication year, author, type of study, country, sample size, laboratory findings. In case of opposite reviewer’s selection, we report both of them or, in view of other publications, we reported those most accredited.

### 2.6. The Assessment of Methodological Quality and Risk of Bias

The funnel-plot and Egger’s regression test were used to assess publication bias [13].

## 3. SARS-CoV-2 Innate Immune System Response

Innate immune system functions protect the host from potential dangerous non-self antigens. For this reason, innate immunity includes different strategy for infection detection and elimination.

In the case of SARS-CoV-2, viral recognition by tissue-resident immune cells within the lung provides a local immune response resulting in the recruitment of further innate immune cells from the blood. This activation is triggered by different SARS-CoV-2 structural components and involves different kinds of immune cells and specific intracellular pathways.

### 3.1. RNA-Sensing of SARS-CoV-2

After host infection, SARS-CoV-2 virus could be recognized through a complex system of sensors, named Pattern Recognition Receptors (PRRs), expressed by epithelial cells as well as by local cells of the innate immune response, such as alveolar macrophages [14]. PPRs recognize specific pathogen portions, called “pathogen-associated molecular patterns” (PAMPs), such as pathogen genome.

The PRRs family includes different components that are involved in the sensing of RNA virus infections. Among these, the most studied referred to SARS-CoV-2 infection sensing, are Toll-Like Receptors (TLRs), and RIG-I-like receptors (RLRs). TLRs consist in a large family of 9 membrane-associated receptors able to recognize different PAMPs [15,16], localized on cell surface (TLR1, TLR2, TLR4, TLR5 and TLR6) or on intracellular membrane (TLR3, TLR7, TLR8 and TLR9). TLRs expressed on cell surface are involved in the recognition of extracellular pathogens, while intracellular TLRs expressed on endosomes and endoplasmic reticulum are engaged during intracellular infection. RLRs intracellular pattern recognition receptors which play a key role in the activation of innate immune system during viral infection. In fact, RLRs are cytoplasmatic RNA helicases involved in the sensing of non-self RNA [17], which include melanoma differentiation-associated protein 5 (MDA-5), retinoic acid-inducible gene I (RIG-I) and the Probable ATP-dependent RNA helicase DHX58 known as LGP2. RLRs are normally inactive in uninfected cells and become active in presence of viral RNA, leading to interferons production to control the infection.

ssRNA genome of SARS-CoV-2 has been reported to be recognized by specific TLRs (TLR3, TLR7, and TLR8), all localized on the endosomal membrane [18,19], and also by MDA-5 and RIG-I, which are able to sense intracellular double-stranded RNA (dsRNA) produced during the infection [17].

**Figure 2:**
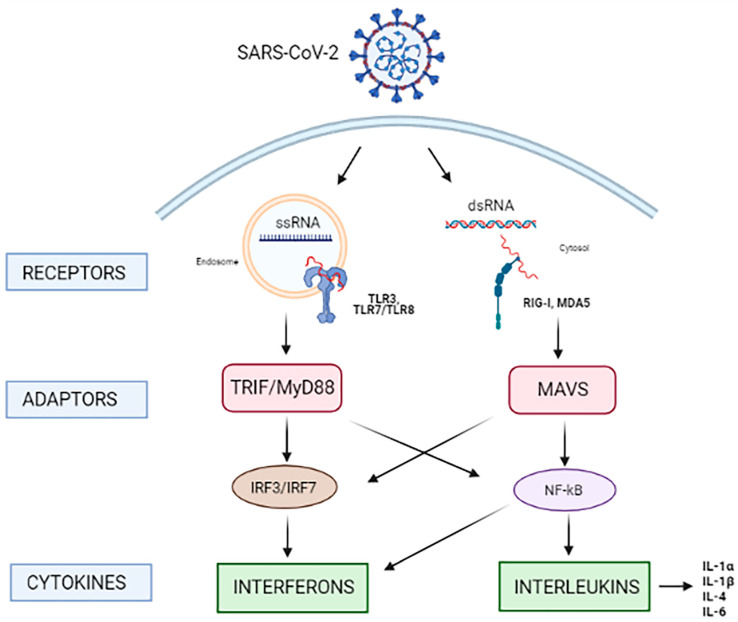
SARS-CoV-2 RNA sensing. After host cells viral infection, SARS-CoV-2 genome is sensed by endosomal Toll-like receptors (TLRs) TLR3, TLR7 and TLR8. TLRs recruit adaptor proteins TIR-domain-containing adapter-inducing interferon-β (TRIF) and myeloid differentiation factor 88 (MyD88). The sensing of replicating virus also occurs by cytosolic RIG-I-like receptors (RLRs) retinoic acid inducible gene I (RIG-I) and melanoma differentiation associated protein (MDA5), that recognize the subgenomic dsRNA of SARS-CoV-2. RIG-I and MDA5 recruit the mitochondrial antiviral signaling protein (MAVS). TLRs and RLRs signaling activate downstream transcription factors, including interferon regulatory factor 3 and 7 (IRF3 and IRF7) and nuclear factor kappa light-chain-enhancer of activated B cells (NF-κB), resulting in production of antiviral interferons and different chemokines (IL-1α, IL-1β, IL-4 e IL-6), which in turn leads to the IRF-3 and IRF-7 phosphorylation required for the expression of IFNs.

After viral RNA binding, both TLRs and RLRs engage adaptor proteins TIR-domain-containing adapter-inducing interferon-β (TRIF) and myeloid differentiation factor 88 (MyD88) are recruited by TLRs, while RIG-1 and MDA5 activate mitochondrial antiviral-signaling protein (MAVS) and consequently recruit specific kinases, such as TANK-binding kinase 1 (TBK1). Then, phosphorylated interferon regulatory factors (IRFs), including IRF3 and IRF7 and transcriptional factors nuclear factor kappa light-chain-enhancer of activated B cells (NF-κB) translocate in the nucleus, inducing the transcriptional activation of genes coding inflammatory cytokines and interferons (IFNs) [9,20,21]. These molecules attract more innate immune cells, such as polymorphonuclear leukocytes, monocytes, NK cells, DC cells, which in turn produce other factors, such as Monokine Induced by Gamma interferon [22], Interferon gamma-induced Protein 10 (IP-10) and Monocyte Chemoattractant Protein 1 (MCP-1), attracting lymphocytes at the site of infection (Figure 2) [9,23,24]. Importantly, TLRs are involved in both innate responses against SARS-CoV-2 infection and in the arise of COVID-19 hyperinflammatory phenotype [17]. Rizzo et al. have recently showed that the activation of TLR3 and TLR7 by SARS-CoV-2 genome differentially involves IRF3 and IRF7, leading to a peculiar production of pro-inflammatory cytokines, such as IL-1α, IL-1β, IL-4, and IL-6, as well as interferons [25]. Furthermore, TLR7 deficient genetic variants has been reported to be associated with a less efficient control of SARS-CoV-2 infection [19].

This role of TLR7 in the antiviral response towards SARS-CoV-2 might represents a potential target for therapy, e.g., with imiquimod, in order to increase TLR7 activation and, consequently, its antiviral effect [26]. Again, alterations of other TLRs, such as TLR2, TLR4 and TLR6, are described associated to excessive inflammation in COVID-19 patients, suggesting the modulation of TLRs as prophylaxis for SARS-CoV-2 infection [19]. 

Both genomic and subgenomic SARS-CoV-2 RNAs are replicated via double-stranded intermediates in the cytoplasm [27]. In this case, RIG-I and MDA5 play a crucial role in the SARS-CoV-2 dsRNA sensing. Taisho et al., reported a peculiar recognition of the 3′ untranslated region of the SARS-CoV-2 RNA genome by RIG-I helicase domains which inhibits the activation of the conventional MAVS-dependent pathways, avoiding cytokine induction. Nevertheless, the interaction of RIG-I with the viral genome directly abrogates viral RNA-dependent RNA polymerase mediation of the first step of replication. These findings suggest the distinctive role of RIG-I as a restraining factor in the early phase of SARS-CoV-2 infection in human lung cells [28]. The crucial role of RLRs in SARS-CoV-2 infection management is also demonstrated by Yang et al., that showed that a deficiency in MDA5, RIG-I or MAVS enhanced viral replication [29].

**Figure 3:**
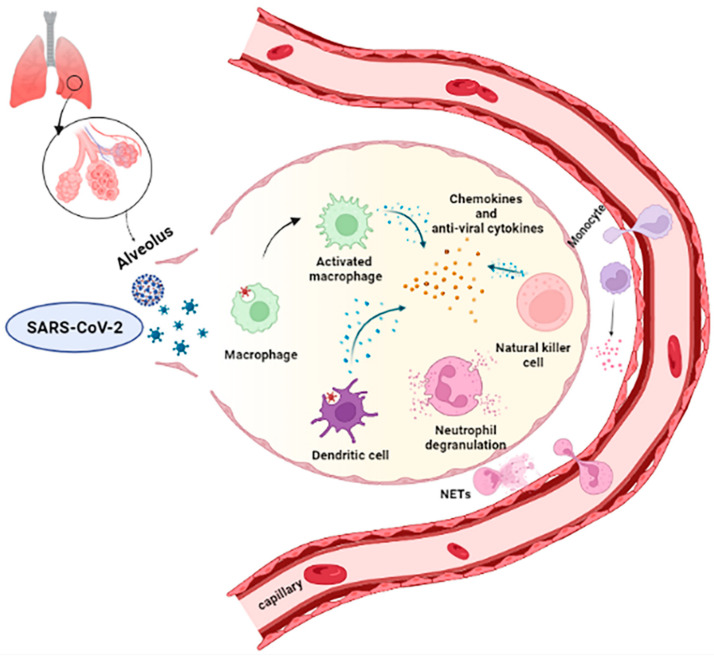
Innate immune response and cytokines storm in SARS-CoV-2 infected lung. The initial viral recognition by tissue-resident immune cells triggers a local innate response. The release of soluble factors, including proinflammatory cytokines and chemokines, from resident immune cells and infected epithelial cells attracts and activates neutrophils, monocytes, macrophages, dendritic cells (DC), natural killers (NK), and innate lymphoid cells into the site of infection, where they contribute to the elimination of the infected cells before virus spreading. This inflammatory environment increased immune cell infiltration from the bloodstream, triggering the “cytokine storm” condition. The hyperinflammation is also sustained by aggregates composed by extracellular DNA fibers, histones, microbicidal proteins, and proteases released from the recruited neutrophils, named also extracellular traps (NETs).

Since the virus could take over by dampening IFNs antiviral effect, the proinflammatory response increases due to the high infiltration of monocytes/macrophages, neutrophils, and several other adaptive immune cells from the bloodstream, resulting in the typical COVID-19 associated “cytokine storms” (Figure 3). 

In addition, the formation of aggregates composed by extracellular DNA fibers, histones, microbicidal proteins, and proteases released from the recruited neutrophils, named also extracellular traps (NETs), causes an hyperinflammatory environment that amplifies the innate immune response, prolonging the recruitments of monocyte/macrophages, neutrophils, NK cells and eosinophils, leading to intensified tissue damage associated with acute respiratory distress syndrome (ARDS) (Figure 3) [30].

As previously discussed, in a significant proportion of infected patients, SARS-CoV-2 induces severe symptoms that are often caused by high levels of pro-inflammatory cytokines which are in part released because of NETs generation, found in more than 80% of neutrophils from COVID-19 patients.

NETs formation follows a multistep process called NETosis, which includes at least three mechanisms: (i) the classical or suicidal NETosis, (ii) the noncanonical pathway, and (iii) the vital NETosis. Although these processes share key components, the required stimuli, the timing and the ultimate result are different [31]. 

In particular, the suicidal NETosis is triggered by the activation of toll-like receptors (TLRs) and complement receptors (CRs) by various ligands [31,32], the noncanonical pathway is stimulated by the lipopolysaccharide (LPS) of gram-negative bacteria, while vital NETosis can be activated by LPS via TLR4, activated platelets, complement proteins, and TLR2 ligands [33]. 

One of the major regulators of NETs formation is the peptidyl arginine deiminase type 4 (PAD4), an important intracellular mechanism of NETosis [34]. PAD4 participates in NETs formation by altering the chromatin status through the cooperation with the neutrophil elastase (NE) and myeloperoxidase [35]. The resulting decondensed chromatin discharged into the extracellular space and leads to neutrophil death, in a process also called NOX-dependent NETosis [36]. 

Since NETs release by neutrophil is mediated by PAD4 activation, these results suggest that in COVID-19 patients circulating neutrophils might be more susceptible to the release of PAD4–dependent NETs, which might cause the systemic increase of soluble NETs observed [37]. 

In addition, in studies performed on murine models of infection and inflammation, the inhibition of PAD4 resulted in reduced NET-associated lung injury [38,39], suggesting the systemic or pulmonary administration of PAD4 inhibitors as a potential treatment for severe COVID-19 [40]. Whilst the idea of blocking NET formation is gaining actraction as a potential therapy for the treatment of severe COVID-19 [40,41,42], this approach could also result in reduced anti-microbial immunity. In fact, although deleterious when generated in excess, NETs play an important role in the entrapment, neutralization and eradication of bacterial and fungal pathogens [43,44] and consequently the use of PAD4 inhibitors could increase the susceptibility of severe COVID-19 patients to secondary infections.

### 3.2. SARS-CoV-2 Inflammasome and Interferon Response

In addition to NETs generation, several studies investigated other possible causes of the characteristic hyperinflammatory environment associated to COVID-19, evidencing that another crucial condition is represented by the ability of SARS-CoV-2 to directly or indirectly activate inflammasomes.

**Figure 4:**
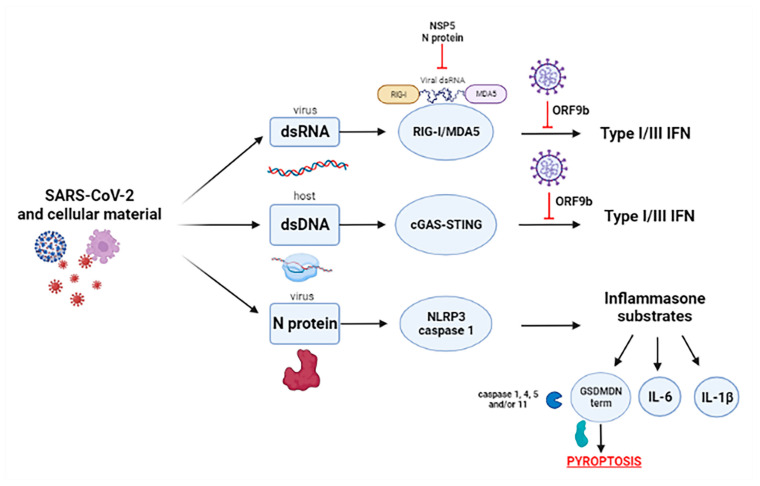
Virus- and host-derived molecules are sensed by several PRRs (Pathogen Recognition Receptors) to induce antiviral and inflammatory responses. SARS-CoV-2 ORF9b and NPS5/N proteins inhibit the activation of type I/III IFNs induced by retinoic acid-inducible gene I (RIG-I)/melanoma differentiation associated protein (MDA5)—mitochondrial antiviral-signaling protein (MAVS) and Cyclic GMP-AMP synthase (cGAS)—Stimulator of Interferon Genes (STING) signaling. SARS-CoV-2 N protein is also able to activate pyrin domain-containing 3 (NLRP3) inflammasome, whose main activation marker is caspase 1, that can lead to the production of IL1B and IL-6 and to pyroptosis, through gastermin D (GSDMD) Nterm cleavage by caspase 1, 4, 5, and/or 11.

In severe manifestations of COVID-19, a massive inflammatory response appears to occur through stimulation of the pyrin domain-containing 3 (NLRP3) inflammasome, that consists in the sensor NLRP3, a NOD-like receptor that interacts with the N-terminus of the adapter protein ASC (also known as PYCARD) via PYD–PYD interactions and an the effector caspase 1, recruited by the caspase recruitment domain (CARD) present in the C-terminus of ASC [45]. The involvement of NLRP3-inflammasome in COVID-19 is confirmed by SARS-CoV-2 N protein possibility to directly interact with NLRP3, thus promoting the inflammasome activation (Figure 4) [46,47].

Once activated, NLRP3 inflammasome causes the release of several proinflammatory cytokines, including IL-6 and IL-1β (Figure 4) [48], which have been reported to have a key role in the pathogenesis of acute lung injury, included COVID-19, affecting type II alveolar epithelial cells ACE2 expression [49,50].

Moreover, NLRP3 activation can lead to pyroptosis, an inflammatory programmed cell death pathway activated through Gasdermin D (GSDMD) cleavage by caspase 1, 4, 5, and/or 11 (Figure 4) that takes place in T lymphocytes and is crucial in the pathogenetic process associated to viral infections [51]. GSDMD triggers pyrotosis by exposing its amino-terminal cell death domain (GSDMDNterm) after caspase cleavage and, in this cleaved form, GSDMD can insert into the cell membrane by binding phosphatidylinositol phosphates and phosphatidylserines, forming pores that kill the cell from within [52,53]. 

Interestingly, even if SARS-CoV-2 infection promotes activation of the NLRP3 inflammasome involving caspase-1, it has been reported that its nucleocapsid proteins are able to inhibit host pyroptosis by blocking GSDMD cleavage [54]. The nucleocapsid binds GSDMD and hinders GSDMD processing by caspase-1, therefore these insights into how SARS-CoV-2 antagonizes cellular inflammatory responses may open new perspectives for COVID-19 treatment.

Although SARS-CoV-2 provokes a pro-inflammatory state, antiviral responses, such as IFNs release, result decreased.

Regarding the pathways driving IFN responses to SARS-CoV-2 infection, it is well known that the virus is a poor type I IFN inducer in vitro [55] and IFN-I analysis on a cohort of 50 COVID-19 patients with various disease severity revealed a highly impaired IFN-I response (characterized by no IFN-β and low IFN-α production and activity), which was associated with a persistent viremia and an exacerbated inflammatory state [56]. However, other findings have shown the presence of neutralizing IFN-I antibodies in critical COVID-19 patients [57] and account for up to 20% of cases of COVID-19 death [58]. 

Among the pathways involved in the altered IFN-I response, RIG-/MDA-5-MAVS and cGAS–STING signaling seem to be particularly involved.

As said above, during SARS-CoV-2 infection, it was expected that the viral ssRNA would be detected by RIG-I and MDA5 RNA sensors, in analogy to other coronaviruses [59]. Despite this evidence, there are numerous findings reporting low amounts and delayed kinetics of these cytosolic RNA sensors, which affect the expression of type I and III IFN in SARS-CoV-2–infected cell lines [60,61]. In particular, SARS-CoV-2 accessory genes ORF9b, an alternative open reading frame within the nucleocapsid (N) gene, has been found to inhibit the activation of types I and III IFNs at mitochondria level [62] by interfering with RIG-I/MDA5-MAVS signaling [63] (Figure 4). SARS-CoV-2 ORF9b also suppressed the induction of types I and III IFNs interfering with TRIF and Stimulator of Interferon Genes (STING) functioin (Figure 4), which are the adaptor protein for the endosome RNA-sensing pathway triggered by TLR3-TRIF and for the cytosolic DNA-sensing pathway involving the Cyclic GMP-AMP synthase (cGAS)-STING signaling, respectively. In particular, SARS-CoV-2 ORF9b inhibits TBK1 phosphorylation induced by both RIG-/MDA-5-MAVS and cGAS–STING signaling and consequently inhibits the phosphorylation and nuclear translocation of IRF3 and types I and III IFN transcription [63].

In addition, both NSP5 and N viral protein disrupted RIG-I-MAVS complex to attenuate the RIG-I-mediated antiviral immunity to affect the IFNs response, while the N protein also affected the recognition of dsDNA by RIG-I (Figure 4) [64].

Concerning cGAS-STING signaling, in both infected cell cultures and COVID-19 patient samples, a specific activation of NF-κB mediated by cGAS-STING recruitment was described (Figure 4) and supported by its attenuation after treatment with several STING-targeting drugs [65]. Moreover, cGAS-STING activity was detected in lung samples of COVID-19 patients with prominent tissue destruction and associated with type I IFN responses. Indeed, a lung-on-chip model revealed that SARS-CoV-2 activates cGAS-STING signaling in endothelial cells through mitochondrial DNA release, leading to cell death and type I IFN production [66].

### 3.3. SARS-CoV-2 Effect on Monocytes and Macrophages

Monocytes and macrophages are antigen presenting cells (APCs) crucial in leucocytes recruitment and inflammation regulation [67] which ensure early responses to pathogens during acute infections.

Alterations of monocyte subset frequency has been reported in inflammatory diseases and infections [68], such as SARS-CoV-2 infection, characterized by low monocyte levels, that gradually increase following recovery [69].

Macrophages consist in a heterogeneous family of phagocytic cells tissue-resident [70]. 

Among these there are lung alveolar macrophages, distinguished into alveolar and interstitial macrophages, which include M1 and M2 macrophages [71]. During SARS-CoV-2 infection, TLR-4, 5, 3, 7 and 9 expressed by macrophages actively sense SARS-CoV-2 N and S proteins and promote M1 polarization of these cells [72].

It has been recently suggested that monocytes/macrophages participate in the onset of cytokine storms observed in COVID-19 patients, and their function seems to be associated to ARDS [73] and poor prognosis [74,75,76,77] in presence of high CCL2 and CCL7 levels [78]. On the other hand, this high percentages of monocyte/macrophages is co-present with lymphopenia. 

**Figure 5:**
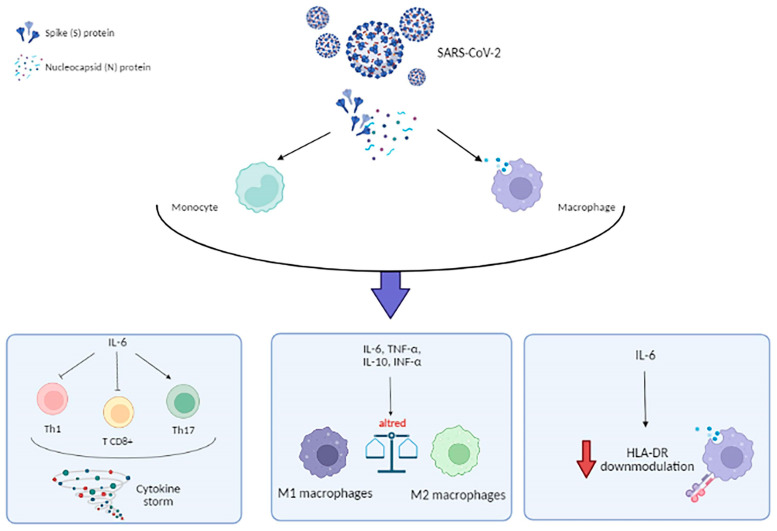
Schematic representation of monocytes/macrophages activation by SARS-CoV-2 Spike (S) and Nucleocapsid (N) proteins and their effect on cytokine profile and Human Leukocyte Antigen-DR (HLA-DR) down-modulation.

Moreover, the expression of viral receptor ACE-2, furin and TMPRSS2 (transmembrane protease serine 2), has been demonstrated in an alveolar mice model [79], suggesting that these cells can be targeted by SARS-CoV-2. Again, monocytes and macrophages culturing in the presence of SARS-CoV-2 S and N proteins resulted in high levels of IL-6 [80] (Figure 5).

In fact, IL-6 plays a central role in SARS-CoV-2-induced cytokine storms associated to ARDS condition [81,82] through Th1 [83,84] and CD8+ T cell inhibition [85] and promotion of Th17 differentiation [86] (Figure 5). 

Typically, SARS-CoV-2 infection firstly stimulates the production of immunoregulatory cytokines by both monocytes and macrophages, and then the virus elicits a transient program dominated by the upregulation of IFNα gene [87]. The most interested cells in the phenotypic change are macrophages, because during viral infection their phenotype has been shown to shifted to an anti-inflammatory of M2 type (Figure 5). 

During SARS-CoV-2 infection, monocytes/macrophages undergo different morphological and phenotypical changes. In particular, a shift from an anti-inflammatory M2 type to an excessive monocyte-macrophage activation is associated to respiratory failure in severe COVID-19 patients [88] (Figure 5), characterized by subsets of mixed M1/M2 macrophage, higher expression of CD80 and CD206 and secretion of IL-6, IL-10, TNF-α, compared to controls [88]. 

As APCs, also SARS-CoV-2 effect on Human Leukocyte Antigen [89] expression on monocyte and macrophage is crucial in the control of the infection. In particular, alteration of specific HLA class II, named HLA-DR, often occurs in response of viral infections [90]. It has been observed that severe COVID-19 present low expression of HLA-DR on monocytes [91] in correlation with ICU (Intensive Care Unit) need (Figure 5) [92,93], probably due to the antagonizing action of IL-6 (Figure 5) [94]. Consequently, HLA-DR decreased expression might be a marker of immune suppression during SARS-CoV-2 infection [91,94].

### 3.4. Role of Neutrophils in COVID-19

Neutrophils are the drivers of hyperinflammation, through degranulation of primary granules and pro-inflammatory cytokines release [95], known to be implicated in COVID-19 pathology [41,96]. Despite their functional protective role, neutrophils extensive and prolonged activation that may occur during SARS-CoV-2 infection, might lead to detrimental effects in the lungs, resulting in cellular infiltrations, ARDS and increased mortality [97]. In fact, high neutrophil count correlate with COVID-19 severity and has been reported to be prognostic marker of ARDS and death [98,99].

Chemokines produced during COVID-19-ARDS recruit neutrophils in the site of infection, supported by transcriptional analysis of bronchoalveolar lavage fluid from patients with high levels of CXCL-2 and CXCL-8 [100,101]. Once recruited in the site of infection, neutrophils release different proinflammatory mediators, including cytokines (interferon-α, interferon-β, tumor necrosis factor, and interleukins 1β, 6, and 10) and chemokine (e.g., CXCL10) that participate in COVID-19 pathogenesis [102]. 

In particular, lung autopsies from patients with ARDS revealed occlusion of pulmonary vessels by NETs, generated by neutrophils-recruitment into alveolar spaces mainly by IL-1b [103] that participate to cytokine overproduction and ARDS [104]. 

Indeed, high levels NETs DNA complexes have been found in serum samples from hospitalized COVID-19 patients, compared to patients with mild/moderate disease and healthy controls [105,106], confirming that the increased infiltration of neutrophil in severe cases contributes to the imbalance of lung’s immune response [95]. 

During neutrophils recruitment to the inflammatory site, endothelial cell-surface molecules have a crucial role, in particular E-selectin and Intercellular Adhesion Molecule-I (ICAM-I). Bortolotti et al. reported that during COVID-19 the expression of these molecules might be modified by HLA-G [35], an immunomodulatory non classical HLA class-I molecule already described associated to COVID-19 condition [107].

### 3.5. SARS-CoV-2 Effect on Natural Killer Cells Activity

NK cells belong to the innate immune system and can recognize pathogens since the early phases of infection. NK cell activation toward infected cells is mainly based on the detection of HLA-I molecule expressed on target cells, that leads to NK cell cytotoxicity when HLA-I is absent. This recognition depends on the engagement of inhibitory and activating NK cell receptors (NKRs) [108], including CD94/NKG2A, CD94/NKG2C, CD94/NKG2E, NKG2D, leukocyte immunoglobulin-like receptors (LILR) and Killer Immunoglobulin-like Receptors (KIR) [109].

It has already been discussed that COVID-19 patients develop an uncontrolled immune response associated with lymphopenia [110,111], showing the reduction of T cell and CD8+ T cell count [112,113], as well as NK cells. Moreover, COVID-19 NK cells have shown a functional exhaustion that, together with their reduced number, have been correlated with the severity of clinical presentation and outcome of the disease [114]. Furthermore, to confirm the key role of NK cells in the outcome of COVID-19, there are some individuals which physiologically have lower expansion and functions of these cells (older patients and immunosuppressed), that exhibited a higher susceptibility to severe and fatal form of COVID-19 [115,116].

The NK cell decrease observed after SARS-CoV-2 infection can be the consequence of both cell death and cell redistribution in infected sites. Xiong et al. showed that several upregulated genes in PBMCs from COVID-19 patients are involved in the apoptosis pathways, suggesting that the peripheral decreased NK cell number may be due to cell-death [100]. By contrast, in favor to the lungs target-site sequestration mechanism, analysis of bronchoalveolar lavage fluid (BALF) samples allowed the detection of higher amounts of NK cells in COVID-19 patients as compared to controls [117]. These data suggest that, upon SARS-CoV-2 infection, NK cells exit the peripheral blood and moves into the lung where they potentially contribute to local inflammation and injury. By contrast, circulating NK cells display an exhausted phenotype that facilitate virus spread to other organs.

**Figure 6:**
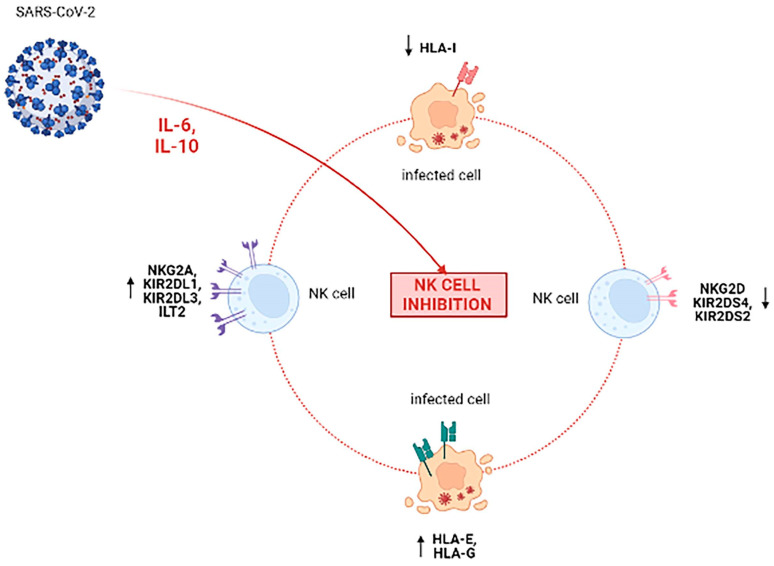
Impairment of NK cells’ functions during SARS-CoV-2 infection. The release of cytokines such as interleukin (IL)-6 and IL-10 affects NK cell activation through changes in NK receptor and HLA molecules expression.

This reduction of NK cells functions is firstly related to local and systemic inflammation. Specifically, elevated IL-6 and IL-10 levels observed in COVID-19, can inhibit NK cytotoxicity, mediated by Granzyme-B production, Fas/FasL (Fas ligand) interaction and CD16 binding with the Fc (constant fraction) of antibodies. Moreover, IL-6 may further impair NK activity by reducing the expression of the activating NKG2D receptor (Figure 6) [118].

The reduced peripheral NK cell count and impaired cytotoxic activity observed in severe SARS-CoV-2-infected subjects, together with the increase of circulating IL-6 levels, suggests that the functional impairment of NK activity leads to enhanced innate immune cell activation with massive proinflammatory cytokine production [119,120].

Another very interesting mechanism of NK cell exhaustion during COVID-19 involving the inhibitory NKG2A receptor has been hypothesized. Zheng et al. observed a significant overexpression of NKG2A receptor (Figure 6) that was decreased simultaneously with the increase in the number of NK cells when the patients were rescued after the infection [112].

The involvement of NKG2A receptor in the mechanisms exploited by SARS-CoV-2 to affect NK cells activation have been investigated by Bortolotti et al. [121]. In this work, authors evaluated the possible effect of SARS-CoV-2 spike proteins (SP) expression by lung epithelial cells on NK cell recruitment and activation [122], reporting that the intracellular expression of SP1 by lung cells reduces the activation of NK cells and their ability to degranulate, identifying SP1 as a causative agent of NK cell function inactivation. 

To better understand the molecular mechanisms exploited by this viral protein in controlling NK cells activation, Bortolotti et al. [121] have enlarged the study to the possible involvement of NK cell receptors and ligands. 

As mentioned above, HLA molecules partly control NK cells via the interaction with their specific NKRs [123] and it has been demonstrated that SP1 is able to specifically up-regulate HLA-E on lung epithelial cells (Figure 6), which is stabilized by SP1-derived HLA-E binding peptide, and at the same time provoked the overexpression of CD94/NKG2A inhibitory receptor levels on NK cells (Figure 6) [121]. These data are in agreement with the recognized crosstalk between HLA-E and CD94/NKG2A, that induces a higher surface level of HLA-E molecules concurrently with a prevalent expression of NKG2A receptor on the surface of NK cells [124].

In addition, individual genetic asset could also contribute to explain the variability in the response of NK cells to SARS-CoV-2. In fact, it has been found out that severe COVID-19 patients showed non-functional or reduced activating receptors (e.g., KIR2DS2) and the prevalence of inhibitory KIRs, in particular of KIR2DL1 and KIR2DL3 (Figure 6) [125,126,127,128], and patients recovering from mild or moderate infection showed the increase of ILT2 inhibitory receptors (Figure 6).

Taken together, these data confirm that patients with severe COVID-19 have a severely compromised innate immune response likely due to a functional exhaustion of peripheral NK cells. Thus, this innate immunity compromission caused by NK cells function exhaustion, is likely to be the direct effect of SARS-CoV-2 infection [129,130]. 

## 4. SARS-CoV-2 Innate Response and Acquired Immunity Cross-Talk

Activation of innate immune system during SARS-CoV-2 infection is crucial in determining the induction of an efficient T and B cell response to obtain specific antibodies secretion and cell-mediated killing of infected cells [131].

**Figure 7:**
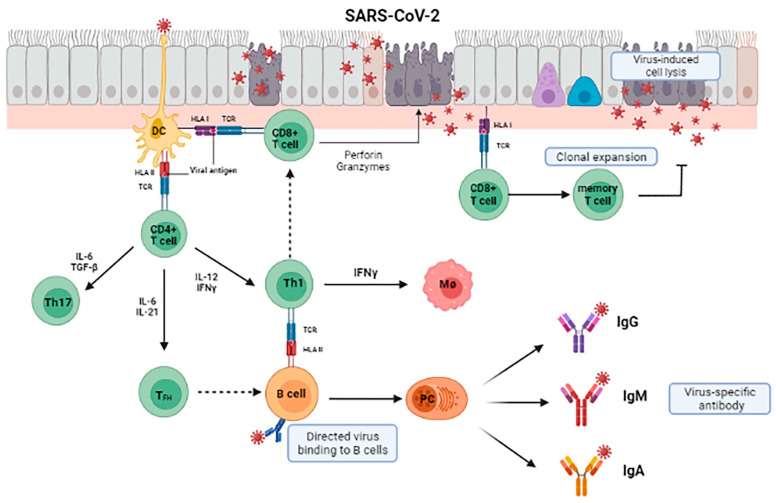
Adaptative immune response during SARS-CoV-2 infection. CD8+ T lymphocytes cytotoxicity is induced by recognition of viral antigens expressed on infected epithelial cells. Subepithelial dendritic cells (DC) recognize present SARS-CoV-2 antigens to CD4+ T lymphocytes, inducing IFN-γ secretion and their differentiation toward memory T helper 1 lymphocytes (Th1), T helper 17 lymphocytes (Th17), and T follicular helper (TFH). TFH helps B cells to develop into plasmacells (PC) and promote the production of IgM, IgA, and IgG isotype SARS-CoV-2-specific antibodies, while Th1, once activated by B cell antigen presentation, activate naïve monocyte (M∅), through IFN-γ secretion.

During SARS-CoV-2 infection, peptides synthetized during viral replication are loaded on HLA class I proteins and presented on the surface of infected cells. The viral peptide-HLA-I complexes recognition by CD8+ cytotoxic T cells induces their activation and expansion [132], leading to the development of virus-specific effector and memory T cells. Moreover, also CD4+ helper T cell, mostly T helper 1 lymphocytes (Th1) and T helper 17 lymphocytes (Th17) [133] recognize SARS-CoV-2 antigens bound by HLA class II (MHLA-II) on professional Antigen presenting cells (APCs) and in turn Follicular helper (FH) T contribute to B cell activation into plasma cells (PC), that release specific anti-SARS-CoV-2 antibodies. At first, IgM are released during the acute phase of infection [134], followed by IgG or secretory IgA, that will also persist as part of immunological memory [135] (Figure 7).

Among APCs, dendritic cells (DCs) represent an important point of junction between innate and adaptive immunity during viral infections. In fact, DCs correct procession and presentation of viral epitopes is fundamental to guarantee a successful B ant T cell priming [136].

The antigenic anatomy of APC/T cell interactions, mediated by HLA-II molecules, is critical to the initiation of productive immune events. In fact, different HLA haplotypes are related to a different susceptibility for distinct disease, including COVID-19. Nguyen et al. [137] sampled the SARS-CoV-2 proteome for interactions with HLA antigens and found that patients characterized by HLA-B*46:01 had the least predicted binding sites for SARS-CoV-2 peptides. However, they also found that the individuals who were HLA-B*15:03 positive showed the highest capacity to bind SARS-CoV-2 peptides. They conclude that individual genetic variations may be critical to the generation of sterilizing immunity to SARS-CoV-2 as well as generation of responses to vaccines. In addition, HLA class I phenotypical variations are important in directing CD8+ T cell responses that mediate cytotoxicity. Poulton et al. [138] found a significant association between HLA-DQB1*06 and SARS-CoV-2 infection risk in transplant patients. A further study in an Italian transplant population, found that HLA-DRB1*O8 showed no peptide binding to SARS-CoV-2 peptides, in association with increased mortality from SARS-CoV-2 [139]. This finding suggests that HLA antigen typing can identify individuals at higher risk for SARS-CoV-2 infection, which could also be ‘super-spreaders’ and at higher risk to develop a severe COVID-19 and poor responses to vaccines [140].

The severe complications associated with SARS-CoV-2 infection encouraged the development of different vaccination strategies. All the vaccine designs, as inactivated and protein subunit vaccines, viral vector vaccines and mRNA vaccines, strongly induce both humoral and cellular specific immunity [141]. For example, SARS-CoV-2 Spike protein encoded by mRNA-based vaccines, once translated and presented by host cells, stimulates Th1, CD8+ T and B cells activation, inducing the production of specific neutralizing antibodies against the virus and normally trigger immune memory, preserving the individual from the developing of the disease. Of course, the production of immunostimulatory epitopes and their efficient presentation by APC are at the basis of a successful immunization. 

The mRNA-based SARS-CoV-2 vaccines elicit antibody responses against the Receptor Binding Domain (RBD) of the spike protein, targeting the same epitopes as occur in natural infection, leading to the production of neutralizing antibodies that target the same epitopes as those produced by natural infection. A study conducted on patients infected by SARS-CoV-2 and subjects vaccinated with Pfizer and Moderna mRNA-based vaccines showed the presence of high titers of IgM and IgG anti-SARS-CoV-2 Spike protein RBD eight weeks after the second injection of vaccine. Moreover, the plasma neutralizing activity and relative numbers of RBD-specific memory B cells of vaccinated volunteers were equivalent to those of individuals who had recovered from natural infection [142,143] and potently neutralize SARS-CoV-2 by targeting a number of different RBD epitopes in common with monoclonal antibodies isolated from infected donors [144,145,146].

## 5. SARS-CoV-2 Immuno-Escaping Mechanisms

As already discussed, innate immune system activation during viral infection leads mainly to IFNs and cytokines production in order to eliminate invading viruses. As other viruses, also SARS-CoV-2 is able to exploit different escape strategies to avoid immune system recognition.

For example, Min et al. [147] have showed how IFN pathway can be a prime target for immune evasion, which could be inhibited by suppressing IFN induction (through decreasing potential PAMPs or disrupting the signaling cascades of IFN induction), function or production. In addition, Kasuga et al. [148] have showed that SARS-CoV-2 proteins, such as nucleocapsid (N) and membrane (M) proteins, are involved in interfering and suppressing IFN signaling [119,149]. 

The recent concern about virus mutations and their effects is further justified by the fact that RNA viruses are characterized by higher mutation rates [150] and SARS-CoV-2 genome alterations are estimated to be 1–2 mutations every month [151]. The genetic diversity of SARS-CoV-2 is the result of errors generated by its RNA-dependent RNA polymerase (RdRp) and recombination [152]. The capacity of coronaviruses to recombine plays a significant role in their evolution and is associated with the strand switching ability of RdRp. As long as a significant number of the world population is infected with SARS-CoV-2, mutations will continue to occur because of the huge number of genome replications and error-prone replication. Therefore, new variants will continue to emerge, and some of them may pose a greater risk for immune escape, being the result of mutations derived by selection based on fitness advantage.

Notably, the main evasive strategy adopted by SARS-CoV-2 is represented by Spike protein mutation acquisition. 

SARS-CoV-2 has different spike protein variants categorized based on their spreading ability, disease severity, immunity, and treatment response. As of 21 October 2021, the European Centre for Disease Prevention and Control (ECDPC) classified variants of concern (VOC), including Beta or B.1.351 (K417N, E484K, N501Y, D614G, A701V), Gamma or P.1 (K417T, E484K, N501Y, D614G, H655Y), and Delta or B.1.617.2 (L452R, T478K, D614G, P681R); variants of interest (VOI), involving Mu or B.1.621 (R346K, E484K, N501Y, D614G, and P681H) and Lambda or C.37 (L452Q, F490S, and D614G); whereas variants under monitoring include various spike protein mutations circulating in different parts of the world [153]. In addition to these, on 26 November 2021 another variant has been designed as VOC [154], which is known as Omicron or B.1.1.529.

In particular, most mutations on SARS-CoV-2 spike protein occur within RBD fragment, especially in the—RBM (S438 to Q506 and K417)—residues involved in ACE2 binding [155,156], suggesting that RBD is the substantial immune-dominance region of SARS-CoV-2 spike protein.

Since all the available vaccination strategies are based on SARS-CoV-2 Spike protein immunization and aim to induce a strong acquired immune response [139], alterations on this protein not only might increase viral tropism and spreading by enhancing its interaction with cellular receptors, but also decreasing vaccines efficiency [89]. 

Furthermore, SARS-CoV-2 evasion of lymphocytes responses has been investigated in vitro, showing that mutations on SARS-CoV-2 Spike and other viral proteins (HLA-I-restricted epitopes) are able to evade in vitro CD8+ T cell responses through abolishing HLA-I binding [157]. 

In addition, in order to reduce HLA-I presentation, SARS-CoV-2 open reading frame 8 (ORF8) is responsible for mediating HLA-I down-modulation by directly interacting with these molecules [158]. The result is that SARS-CoV-2–infected cells by expressing ORF8 are much less sensitive to lysis by cytotoxic T lymphocytes and evade immune surveillance, due to the antigen presentation system impairment caused by this viral protein.

Moreover, SARS-CoV-2 can also infect regulatory T (Treg) cells through the binding of NRP1 [159], another coreceptor of SARS-CoV-2, thereby reducing Treg population and leading to uncontrolled host proinflammatory responses.

Again, SARS-CoV-2 has developed several strategies to escape innate immune system, based on TLRs and RLRs sensing interference by several viral proteins, thus affecting cytokine and interferon production (Figure 3) [9,147,148].

## 6. Conclusions/Perspectives

The central role of host innate immune system during SARS-CoV-2 infection is under investigation. Since innate immunity is involved in the earliest stages of SARS-CoV-2 infection, its correct activation is necessary to guarantee an efficient control of viral spread. The primary antiviral effect exerted by innate immune system, based mainly on interferons production and direct killing of infected cells by NK cells, cytokines secretion and a correct antigens presentation, is strictly connected to the activation of T and B lymphocytes. In view of this, the identification of the strategies able to improve the innate immune response towards SARS-CoV-2 infection might represent an advantage not only to control the natural infection, but also to increase the active immunization by vaccination. There are several courses of action that can be undertaken to effectively subdue the pandemic. At first, it would be necessary to closely monitor the emergence of novel SARS-CoV2 variants globally. Secondly, vaccination protocols should be adjusted to current variants. Finally, an effort should be made to develop therapeutic protocols involving monoclonal antibodies in order to offer reliable protection against emerging variants.
